# Efficacy of Topical Compound Danxiong Granules for Treatment of Dermatologic Toxicities Induced by Targeted Anticancer Therapy: A Randomized, Double-Blind, Placebo-Controlled Trial

**DOI:** 10.1155/2017/3970601

**Published:** 2017-08-06

**Authors:** Aiping Tian, Aiping Zhou, Xinyu Bi, Shangying Hu, Zhichao Jiang, Wen Zhang, Zhen Huang, Hongzhe Shi, Boyan Yang, Wei Chen

**Affiliations:** ^1^Department of Traditional Chinese Medicine, National Cancer Center, Cancer Hospital, Chinese Academy of Medical Sciences and Peking Union Medical College, Beijing 100021, China; ^2^Department of Medical Oncology, National Cancer Center, Cancer Hospital, Chinese Academy of Medical Sciences and Peking Union Medical College, Beijing 100021, China; ^3^Department of Hepatobiliary Surgery, National Cancer Center, Cancer Hospital, Chinese Academy of Medical Sciences and Peking Union Medical College, Beijing 100021, China; ^4^Department of Cancer Epidemiology, National Cancer Center, Cancer Hospital, Chinese Academy of Medical Sciences and Peking Union Medical College, Beijing 100021, China; ^5^Department of Urology, National Cancer Center, Cancer Hospital, Chinese Academy of Medical Sciences and Peking Union Medical College, Beijing 100021, China; ^6^Emergency Department, National Cancer Center, Cancer Hospital, Chinese Academy of Medical Sciences and Peking Union Medical College, Beijing 100021, China; ^7^Department of Pharmacy, National Cancer Center, Cancer Hospital, Chinese Academy of Medical Sciences and Peking Union Medical College, Beijing 100021, China

## Abstract

Dermatologic toxicities resulting in dose reduction or discontinuation of treatment pose challenges for targeted anticancer therapies. We conducted this randomized, double-blind, placebo-controlled trial to investigate the efficacy of topical application of Compound Danxiong Granules (CDG) for treatment of dermatologic toxicities associated with targeted anticancer therapies. One hundred and ten patients with dermatologic toxicities induced by targeted anticancer therapies were randomly assigned to CDG or placebo group. Each crude herb (*Rhizoma Chuanxiong, Paeonia suffruticosa *Andr.,* Cortex Phellodendri, Geranium sibiricum *L., and* Flos Carthami*) was prepared as an instant herbal powder. Application of the CDG via topical washes lasted 20 minutes, twice daily, for 10 days. The primary outcome was the total effective rate, defined as reduction in at least one grade of skin toxicity. The total effective rate was 77.61% (52/67) in the CDG group and 27.27% (9/33) in the placebo group (*P* < 0.0001). Compared to the placebo treatment, CDG treatment achieved a higher total effective rate for hand-foot skin reaction (95.45% versus 27.27%), acneiform eruption (69.23% versus 30.78%), and paronychia (68.42% versus 22.22%). Topical application of CDG can effectively attenuate dermatologic toxicities induced by targeted anticancer therapies. The effect of CDG was more pronounced in hand-foot skin reaction.

## 1. Introduction

Targeted anticancer therapies have improved survival outcomes in various cancer patients [1]. Anticancer agents target signal transduction, tumor angiogenesis, or tumor microenvironment pathways [2]. Introduction of these agents has been associated with a wide spectrum of dermatologic toxicities [3]. Cutaneous adverse reactions associated with targeted anticancer therapies commonly manifest as acneiform eruption, hand-foot skin reaction, paronychia, skin fissures, and dermal hypersensitivity reaction [4]. Despite the fact that these dermatologic toxicities are not life threatening, dose reduction or discontinuation of cancer treatment and impairments in patient quality of life pose a great challenge for targeted cancer therapies [5]. Consequently, management of these dermatologic toxicities further increases the economic burden of cancer care [6]. However, mechanisms underlying the targeted anticancer therapies-associated cutaneous toxicities remain incompletely characterized. Interference with the follicular and interfollicular epidermal growth signaling pathway is thought to be critical [7]. Therefore, unified standardized therapy for management of the targeted anticancer therapies associated with dermatologic toxicities is lacking [8]. Currently, the primary therapies to treat dermatologic symptoms are antibody, antiallergic, and local supportive treatments [9], but these agents are not highly effective. Therefore, there is an urgent need to find an alternative therapeutic agent to prevent and manage dermatologic toxicities induced by targeted anticancer therapies.

Traditional Chinese Medicine (TCM) has long been used for the treatment of dermatologic disorders [10, 11] and cancer [12, 13]. Thus, consideration should be given to the beneficial effects of Chinese herbal prescription for management of dermatologic toxicities induced by targeted anticancer therapies. Many TCM practitioners have attempted to use Chinese herbs for management of targeted agent-related dermatologic toxicities [14]. Topical applications are the most accessible type of herbal treatment. However, evidence of topical use of Chinese herbal prescription in management of dermatologic toxicities induced by anticancer therapies was mainly based on case series, physician experiences, or self-controlled studies. Thus, there is a lack of well-designed double-blind, placebo-controlled trials to support the evidence.

The objective of this trial was to investigate the efficiency of Compound Danxiong Granules (CDG) in the treatment of dermatologic toxicities induced by targeted anticancer therapies.

## 2. Methods

### 2.1. Study Design and Patients

This randomized, double-blind, placebo-controlled trial was conducted in the Department of Traditional Chinese Medicine at the National Cancer Center/Cancer Hospital, Chinese Academy of Medical Sciences and Peking Union Medical College, from September 2010 to July 2014. The Ethics Committee of Cancer Hospital, Chinese Academy of Medical Sciences, approved the study protocol. Written informed consent was obtained from all patients before enrollment. Patient eligibility was based on the following inclusion criteria: (1) aged 18 years or over; (2) an Eastern Cooperative Oncology Group performance status ≤ 3; (3) malignant tumor diagnosed by evidence of pathological or cytological findings; (4) treatment with targeted anticancer agents and occurrence of dermatologic toxicities including acneiform eruption, paronychia, and hand-foot skin reaction; and (5) not receiving other preparations of traditional herbal medicines during the 4 weeks before enrollment. The exclusion criteria were (1) dermatologic toxicities not induced by targeted anticancer agents; (2) concurrent acne vulgaris, eczema, psoriasis, and other skin diseases; (3) skin hypersensitiveness; and (4) intellectual and mental disorders, abnormal language expression ability, and an inability to judge or express their own symptoms.

### 2.2. Preparation of CDG and Placebo

This study utilized five Chinese herbs:* Paeonia suffruticosa *Andr. (20 g),* Rhizoma Chuanxiong* (18 g),* Flos Carthami* (12 g),* Cortex Phellodendri* (18 g), and* Geranium sibiricum *L. (20 g). The detailed characteristics of the constituents in CDG are summarized in Table 1. All crude herbs were purchased from China Resources Sanjiu Medical & Pharmaceutical Co., Ltd., and were prepared as an instant herbal powder. The cotton bag containing the above instant herbal powder was immersed in 60–70°C water and then diluted in 1,000 ml water. The bag was stirred to accelerate release of the effective ingredients until the temperature dropped to 25–30°C. Placebo powder was made from dextrin and edible pigment and had the same color and appearance as CDG.

### 2.3. Intervention

Eligible patients were randomly allocated 2 : 1 to either a CDG (*n* = 73) or placebo (*n* = 37) group using a computer-generated randomization code. The investigators were blinded to the sequence code until the trial was completed. The tested agents were supplied in labeled packaged containers and provided to the patients and the investigators in a double-blind manner. Patients were instructed to use their prescribed preparation before treatment. Patients were asked to start topical application of their tested drugs after random grouping. For hand-foot skin reaction and paronychia patients, the skin lesions were soaked in the herbal liquid. For acneiform eruption in the facial and trunk cadre patients, a gauze patch with herbal liquid was repeatedly applied to the skin lesions. A dosage of CDG and placebo was used as a topical wash for 20 minutes, twice daily, until the water temperature dropped to below 25°C. Treatment duration lasted for 10 consecutive days.

### 2.4. Assessment of Dermatological Toxicities

Rash severity was graded according to the US National Cancer Institute Common Toxicity Criteria for Adverse Events version 3.0 (NCI CTCAE v3.0) [15] and by consensus among Chinese oncologist experts: Grade I: localized rash (mainly face and upper trunk) without associated symptoms, without interfering with daily activities, and with no signs of secondary infection; Grade II: scattered, but not generalized eruption, slightly interfering with daily activities, and without secondary infection signs; and Grade III: widespread rash with severe objective symptoms, interfering with daily activities, and associated with local secondary infection. The severity of nail changes was graded according to the NCI CTCAE v3.0: Grade I: nail decolorization and wrinkles, without disruption of daily activities; Grade II: nail partially or completely detached and painful nail plate; and Grade III: severe nail plate lesion (paronychia) interfering with daily activities and/or secondary infection. Hand-foot skin reaction was also graded in accordance with the NCI CTCAE v3.0: Grade I: minimal skin changes or dermatitis (e.g., erythema and scaling) without pain; Grade II: skin changes (e.g., peeling, blisters, bleeding, and edema) or pain, not interfering with daily activities; and Grade III: ulcerative dermatitis or skin changes with pain interfering with daily activities.

### 2.5. Outcome Measures

The primary end point was the total effective rate. “Effective” indicated a reduction in at least one grade in dermatological toxicity. “Cure” was defined as the absence of skin lesions. “Treatment failure” indicated no improvement in grade of dermatological toxicity. The total effective rate was defined as the number of patients who achieved “Effective” (reduction in at least one grade of dermatological toxicity) divided by the total number of patients. The secondary end point was safety determined by routine blood tests and measurements of liver and renal function. Physicians were trained to grade dermatitis before the beginning of this trial. Dermatological toxicities were evaluated at baseline and at the end of treatment by experienced oncologists and dermatologists. All investigators took photographs using a CANON Digital IXUS 500 at enrollment and after 10 days of treatment. Photographs were used with patient authorization. Routine blood tests and liver and renal function were determined before and after 10 days of treatment. The following data were also collected for each patient: type of tumor, targeted anticancer agents, skin lesion type, severity of skin lesions, and ongoing, targeted anticancer therapy.

### 2.6. Statistical Analysis

The sample size was calculated to ensure specific significance, during which statistical power was taken as 90% at the 5% significance level. Statistically significant increases in the total effective rate at 25% were used for this purpose based on our pilot study comparison of the total effective rate of the CDG group (60%) and the control group (35%). Finally, considering a 10% dropout rate, our study required a total of 111 patients. All statistical analyses were carried out using SPSS 19.0 software (SPSS Inc., Chicago, IL, USA). Categorical data are expressed as frequencies and percentages. Comparisons of categorical variables between the CDG and placebo groups were analyzed using the Chi-square test. The Fisher exact test was utilized. A *P* value < 0.05 was considered statistically significant.

## 3. Results

### 3.1. Baseline Characteristics

**Figure 1:**
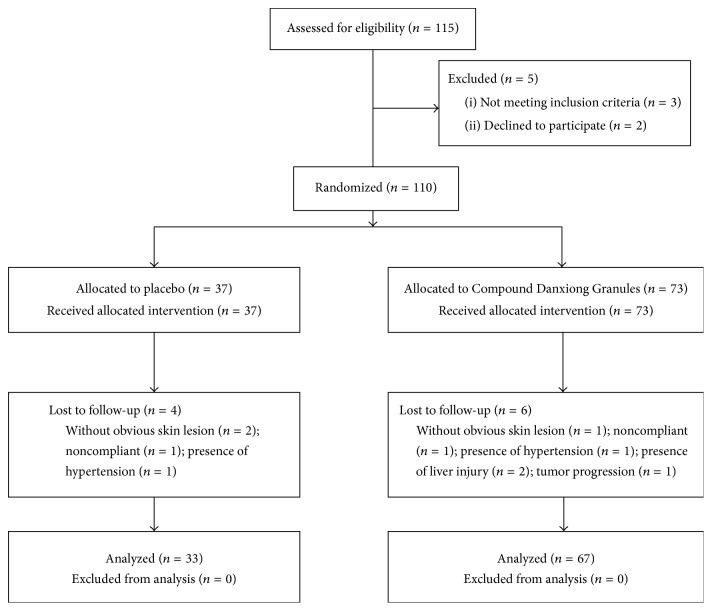
Flow diagram of patient enrollment and randomization.

Figure 1() shows the flow diagram of patient enrollment and randomization through the study. A total of 110 eligible patients were randomized and 100 patients completed planned topical wash therapy and assessment. Ten patients discontinued topical washing treatment. The reasons for discontinuation of topical washing treatment were spontaneous pain relief (*n* = 2) and being lost to follow-up (*n* = 3). Discontinuation of anticancer treatment resulted from severe hypertension, liver injury, or disease progression (*n* = 5).  Table 2 shows the baseline characteristics of the two treatment groups. The most frequently reported skin toxicities among the included patients were acneiform eruption (39.0%), hand-foot skin reaction (33.0%), and paronychia (28.0%). There was no significant difference between the two groups with regard to age or gender distribution, tumor type, type of skin lesions, targeted anticancer agents, and severity of skin lesions (all *P* > 0.05).

### 3.2. Comparison of Total Clinical Effect

As shown in Table 3, treatment was effective in 52 (77.61%) cases in the CDG group and 9 (27.27%) cases in the placebo group, with a statistically significant difference between the two groups (Chi-square = 23.55, *P* < 0.001). The clinical cure rate was 12/67 patients (17.91%) in the CDG group and no patients achieved a clinical cure in the placebo group.

### 3.3. Comparison of the Clinical Effect on Different Types of Skin Lesions

**Figure 2:**
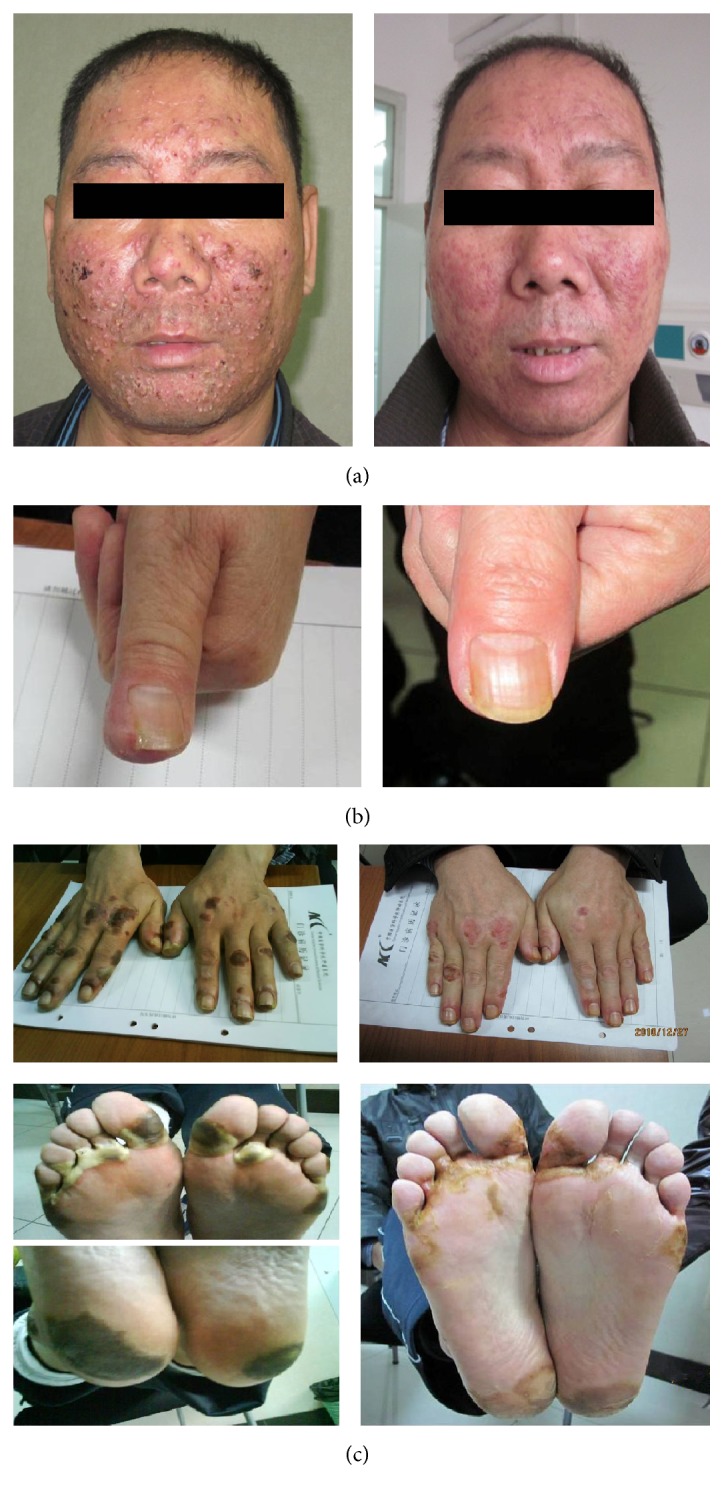
Three representative patients before and after treatment. (a) Acneiform eruption associated with Erlotinib in a patient with lung cancer. Before treatment (left), the face displayed swelling, itching, pain, purulent heads, and hemorrhage (Grade III). After 10 days of topical washes with CDG (right), the rash nearly healed. (b) Paronychia associated with Erlotinib in a patient with lung cancer. Before treatment (left), the nail exhibited pain, redness, and swelling (Grade III). After 10 days of topical washes with CDG (right), the tissues surrounding the nail recovered. (c) Hand-foot skin reaction associated with Sorafenib in a patient with hepatocellular carcinoma. Before treatment (left), skin areas on both hand and foot displayed redness, swelling, foaming, bleeding blister, and pain (Grade III). After 10 days of topical washes with CDG (right), the lesions were nearly healed.

As shown in Table 4, the treatment for hand-foot skin reaction, acneiform eruption, and paronychia was effective in 21 (95.45%), 18 (69.23%), and 13 (68.42%) cases in the CDG group, respectively. In contrast, the treatment for hand-foot skin reaction, acneiform eruption, and paronychia was effective in 3 (27.27%), 4 (30.78%), and 2 (22.22%) cases in the placebo group, respectively. There were statistically significant differences regarding hand-foot skin reaction, acneiform eruption, and paronychia between the two groups (*P* < 0.05). The clinical cure rate in CDG treated patients was 31.82% for hand-foot skin reaction, 11.54% for acneiform eruption, and 10.53% for paronychia. Before and after CDG treatment, images are presented in Figure 2().

### 3.4. Incidence of Discontinuation of Cancer Treatment

Discontinuation of cancer treatment was reported in 6 (9.96%) patients in the CDG group and 4 (12.1%) patients in the placebo group. There was no significant difference with regard to the incidence of discontinuation of cancer treatment between the two treatment groups (*P* = 0.887). Discontinuation of targeted anticancer therapy was not correlated with skin toxicity. Adjustment of targeted anticancer agents and/or liver or cardiovascular toxicities caused by the targeted therapies were reasons for discontinuation of cancer treatment.

### 3.5. Adverse Effects

The skin treatment regimen was well tolerated and did not cause any adverse effects. The routine blood tests and biochemical analyses did not differ when comparing before and after herbal treatment.

## 4. Discussion

This randomized, double-blind, placebo-controlled trial demonstrated that topical use of CDG, as an instant herbal powder, was significantly effective in minimizing dermatologic toxicities induced by targeted anticancer therapies. More importantly, the cure rate (31.82%) and total effective rate (95.45%) of hand-foot skin reaction were more pronounced in CDG treated patients compared to patients in the placebo group.

In this study, the incidence of dermatologic toxicities was 31% for Cetuximab, 20% for Sorafenib, and 18% for Erlotinib. The most frequently encountered dermatologic toxicities were acneiform eruption (39%), hand-foot skin reaction (33%), and paronychia (28%). The total effective rate in the CDG group was significantly higher than that of the placebo group (77.61% versus 27.27%, resp.). The effect of CDG on hand-foot skin reaction was particularly more pronounced, and nearly one-third of these patients with Grade II or higher skin lesions achieved clinical cure. The painful symptoms and skin lesions were attenuated during days 3–10 following treatment.

There are many approaches for the management of dermatologic toxicities induced by targeted anticancer therapies [9]. Early intervention is critical in treating dermatologic toxicities. Appropriate and timely management of dermatologic toxicities may prevent dose alteration or discontinuation of cancer treatment. A well-designed systematic review indicated that antibiotics were the most common treatment option and had the potential to reduce the severity of epidermal growth factor receptor inhibitor-induced skin toxicities [16]. Unfortunately, a unified standard therapy based on these studies is still lacking.

Traditional Chinese herbal therapies have demonstrated benefits for management of dermatologic toxicities induced by targeted anticancer agents [17]. An early randomized controlled trial (RCT) [18] showed that antipruritic prescription* (Radix Stemonae, Xanthium sibiricum, clove, lotus leaf, honeysuckle, Sophora flavescens *Ait.*, Portulaca oleracea, and dwarf lilyturf)* was superior to calamine lotion in attenuating acneiform eruption caused by targeted anticancer agents. Another RCT [19] demonstrated that external application of Zhiyang Pingfu Liquid significantly attenuated itching, rash, and acneiform eruptions induced by epidermal growth factor receptor inhibitors compared with erythromycin ointment. Zhiyang Pingfu Liquid formula is composed of* Scutellaria baicalensis, Sophora flavescens, Portulaca oleracea, and bark of Dictamnus dasycarpus*. Zhang et al. [20] investigated the clinical efficacy of the Zhiyang Pingfu Liquid formula as an oral treatment combined with topical administration of Yangfei Xiaozhen Formula (*coastal glehnia root, Ophiopogon japonicus, Schisandra chinensis, honeysuckle, Sophora flavescens, bark of Dictamnus dasycarpus, cortex moutan*, etc.) for treating rash induced by lung cancer targeted agents in 80 patients. They found that the total curative effect of rash grade was 75% in the patients receiving Yangfei Xiaozhen Formula combined with external therapy and 55% in the patients receiving external therapy alone (*P* < 0.05). Moreover, oral Yangfei Xiaozhen Formula significantly improved the quality of life compared to the control group.

Chinese herbal therapies for dermatologic toxicities can be divided into two types of oral and topical medicines. Topical use is the most commonly prescribed intervention for dermatologic patients. Topically applied herbal lotion lowers the first pass effect through the liver and degradation in the gastrointestinal tract. Economically, the tested herbs in this trial are generally low in cost. More importantly, topical application of CDG effectively attenuated multiple dermatologic lesions associated with hand-foot skin reaction, acneiform eruption, and paronychia. Furthermore, since CDG is an instant herbal powder, it can be easily applied by all health care professionals. For these reasons, CDG may be considered for patients with targeted anticancer therapy-related dermatologic toxicities. However, the exact mechanisms underlying CDG's effect on reducing dermatologic toxicities are largely unknown. Based on TCM theory, CDG formula works on the principles of clearing heat and draining dampness, cooling, activating blood, and resolving stasis. Taken together, the above herbs could produce a beneficial effect on patients who experience dermatologic toxicities.

Some potential limitations in the current study should be noted. First, an important limitation is the short period of intervention. The relatively lower effect of CDG on acneiform eruption and paronychia may be correlated with shorter duration of intervention. A 10-day topical intervention may not be long enough for recovery of acneiform eruption and paronychia. Future studies should extend the treatment and follow-up duration. Second, our trial did not observe the preventive effect of CDG. As for the high prevalence of targeted anticancer therapy associated skin lesions (60–80%), CDG may provide a useful role in prevention of skin toxicities, thereby increasing patient compliance to targeted anticancer therapies.

## 5. Conclusions 

Our study suggests that topical application of CDG can effectively attenuate dermatologic toxicities associated with targeted anticancer therapy. The treatment effect of CDG was more pronounced in hand-foot skin reaction. A well-designed RCT with large sample sizes is required to confirm the preventive effect of CDG in targeted anticancer therapy associated skin lesions.

## Figures and Tables

**Table 1:** Constituents in the Compound Danxiong Granules.

Chinese name	English herb name	Latin herb name	Family	Species	TCM action	Dose used
Chuān xiōng	Szechuan lovage Rhizome	*Rhizoma Chuanxiong*	Apiaceae	*Ligusticum chinense* (L.) Crantz	Blood-activating	18 g
Mǔ dān pí	Tree Peony Bark	*Paeonia suffruticosa *Andr.	Paeoniaceae	*P. suffruticosa*	Cooling the blood and detoxicating	20 g
Huáng bò	Amur Corktree Bark	*Cortex Phellodendri*	Rutaceae	*Phellodendron chinense* Schneid.	Clearing heat and dampness	18 g
Lǎo Guàn Cǎo	Cranesbill Herb	*Geranium sibiricum *L.	Geraniaceae	*Geranium*	Clearing dampness and blood-activating	20 g
Hóng huā	Safflower	*Flos Carthami*	Compositae	*Carthamus tinctorius* L.	Blood-activating	12 g

**Table 2:** Baseline patient characteristics.

Characteristic	Compound Danxiong Granules (*n* = 67)	Placebo (*n* = 33)	*P* value
Mean age/range (years)	55 (31–72)	53 (33–70)	0.478
Sex			0.841
Male	44 (65.7%)	21 (63.6%)	
Female	23 (24.3%)	12 (26.4%)	
Type of tumor			0.992
Hepatocarcinoma	12 (17.9%)	5 (15.2%)	
Renal carcinoma	10 (14.9%)	6 (18.2%)	
Lung cancer	22 (32.8%)	11 (33.3%)	
Intestinal cancer	21 (31.3%)	10 (30.3%)	
Breast cancer	1 (1.5%)	1 (3.0%)	
Soft tissue sarcoma	1 (1.5%)		
Targeted anticancer agents			0.997
Sorafenib	14 (20.9%)	6 (18.2%)	
Sunitinib	8 (11.9%)	5 (15.2%)	
Erlotinib	12 (17.9%)	6 (18.2%)	
Gefitinib	10 (14.9%)	5 (15.2%)	
Cetuximab	21 (31.3%)	10 (30.3%)	
Famitinib	2 (3.0%)	1 (3.0%)	
Type of skin lesions			0.994
Hand-foot skin reaction	22 (32.8%)	11 (33.3%)	
Acneiform eruption	26 (38.8%)	13 (39.4%)	
Paronychia	19 (28.9%)	9 (27.3%)	
Severity of skin lesions			0.134
Grade I	5 (7.5%)	6 (18.2%)	
Grade II	28 (41.8%)	16 (48.5%)	
Grade III	34 (50.7%)	11 (33.3%)	
Time after the initiation of targeted anticancer therapies	23 ± 10 (days)	24 ± 14 (days)	0.700

**Table 3:** Comparison of clinical effective rate between treatment groups.

Group	Total effective rate	Treatment failure rate	Chi-square	*P* value
Compound Danxiong Granules (*n* = 67)	52 (77.61%)	15 (22.39%)	23.55	<0.0001
Placebo (*n* = 33)	9 (27.27%)	24 (72.73%)		

**Table 4:** Comparison of clinical effective rate on different types of skin lesions.

Group	Total effective rate	Treatment failure rate	*P* value
Hand-foot skin reaction			<0.0001
Compound Danxiong Granules (*n* = 22)	21 (95.45%)	1 (4.55%)	
Placebo (*n* = 11)	3 (27.27%)	8 (72.73%)	
Acneiform eruption			0.039
Compound Danxiong Granules (*n* = 26)	18 (69.23%)	8 (30.77%)	
Placebo (*n* = 13)	4 (30.78%)	9 (69.23%)	
Paronychia			0.042
Compound Danxiong Granules (*n* = 19)	13 (68.42%)	6 (31.58%)	
Placebo (*n* = 9)	2 (22.22%)	7 (77.78%)	
